# Renal Protective Mechanisms of Shenyuan Particle in Db/Db Mice: A Study Based on Network Pharmacology

**DOI:** 10.1155/2022/9579179

**Published:** 2022-06-14

**Authors:** Guoshuang Zhu, Lan Wang, Zenan Wu, Mingliang Qiu, Shiwen Ke, Liangji Liu, Xiaoqin Wang

**Affiliations:** ^1^Jiangxi University of Chinese Medicine, Nanchang 330004, China; ^2^Hubei Hospital of Traditional Chinese Medicine, Wuhan 430070, China; ^3^Affiliated Hospital of Hubei University of Traditional Chinese Medicine, Wuhan 430070, China; ^4^Hubei Academy of Traditional Chinese Medicine, Wuhan 430070, China; ^5^Hubei University of Chinese Medicine, Wuhan 430065, China; ^6^Affiliated Hospital of Jiangxi University of Chinese Medicine, Nanchang 330006, China

## Abstract

**Aim:**

The renal protective mechanisms of Shenyuan particle (SYP) in the treatment of diabetic kidney disease (DKD) were investigated, focusing on the main targets and pathways.

**Materials and Methods:**

In this study, the potential targets of compounds identified in SYP were predicted by Traditional Chinese Medicine Systems Pharmacology Database and Analysis Platform (TCMSP), and a “herb-compound-target” network was constructed via Cytoscape. Next, the Gene Ontology (GO) terms and Kyoto Encyclopedia of Genes and Genomes (KEGG) functional enrichment analyses were dissected using R language. A protein-protein interaction network was fabricated using STRING to obtain the main target information. In addition, db/db mice were used as the DKD models to explore the renal protective effects of SYP. Transmission electron microscopy, western blot, pathological staining, TUNEL staining, and biochemical methods were used to identify the apoptotic pathways and establish the primary mechanism of SYP.

**Results:**

Network pharmacology analysis revealed 67 potential targets based on the analysis of different databases. The targets of SYP were primarily associated with apoptosis. The network hub genes included caspase 3, caspase 7, caspase 8, caspase 9, Bax, and Bcl-2. In vivo, SYP materially improved renal function and inhibited apoptosis in the db/db mouse kidneys by improving the mitochondrial health. In addition, our results showed that SYP significantly decreased the expression of Bax, caspase 3, and Cyto-c and increased the expression of Bcl-2.

**Conclusions:**

Network pharmacology analysis and experimental results suggest that SYP ameliorates DKD mediated via multiple components, targets, and pathways. Our study further demonstrates that SYP inhibits apoptosis in the kidneys of db/db mice by improving the mitochondrial health and thereby alleviating renal damage.

## 1. Introduction

According to the International Diabetes Federation Diabetes Atlas 9^th^ edition, an estimated 1.25 billion individuals worldwide will be diagnosed with diabetes by 2030, increasing to 1.50 billion by 2045 [1]. With the increasing prevalence of diabetes mellitus (DM), the incidence of diabetic kidney disease (DKD) is also increasing worldwide [2]. Currently, DKD is a serious microvascular complication of DM. In the latest study, 40% of the patients progressed to DKD [3]. DKD is the primary cause of end-stage renal disease (ESRD), which increases the death rate of patients with DM [4]. Currently, it is not possible to delay the progression of DKD to ESRD effectively by controlling blood glucose levels and blood pressure as well as blockade of the renin-angiotensin-aldosterone system [5]. Therefore, there is an urgent need for innovative drugs to treat DKD. Studies have shown that traditional Chinese medicine (TCM) was successfully used to treat DKD, with interventions such as berberine [6], Qidan Dihuang grain (QDDHG) [7], and Danggui Shaoyao San (DSS) [8]. TCM is characterized by multicomponent, multitarget, and multichannel approaches, such as regulation of glucose metabolism [9], treatment of lipid metabolism disorder [10], inhibiting the activation of polyol pathway [11], antioxidative stress interventions [12], improving the structure and function of podocytes [13], and inhibiting inflammatory response [13].

The core idea of network pharmacology is strongly consistent with the “holistic view” of TCM. It provides insight into the efficacy and mechanism of multi-ingredient TCM from a holistic perspective. In a previous study [14], Shenyuan Particle (SYP), a TCM product, was used for the treatment of DKD mice, which alleviated kidney damage. To elucidate the underlying mechanisms of SYP in DKD, this study developed a network prediction model of TCM to predict the potential drug targets. Potential targets and pathways of SYP were determined using a herb-compound-target interaction network. The db/db mice were used as DKD models to validate the protective effects and mechanisms of SYP.

## 2. Materials and Methods

### 2.1. Target Delineation

SYP comprises *Radix Astragali*, *Rhubarb*, and *Herba Epimedii*. In this study, the protein targets of this compound were retrieved from the Traditional Chinese Medicine Systems Pharmacology Database and Analysis Platform (TCMSP) (http://lsp.nwu.edu.cn/tcmsp.php), a systematic pharmacological analysis platform of TCM, and GeneCards database. Based on the pharmacokinetic parameters of ADME, the components with an oral bioavailability (OB) ≥30% and a drug likeness (DL) ≥0.18 was defined as active ingredients. OB represents the pharmacological percentage of an oral drug entering the systemic circulation. A high OB is usually the key index to determine the bioactive molecules in therapeutic drugs. DL is a qualitative concept used to estimate the pharmacological properties of compounds, which enables optimization of the pharmacokinetic and drug properties, such as solubility and chemical stability. Subsequently, candidate compounds were incorporated into the TCMSP to identify the related protein targets.

### 2.2. Protein-Protein Interaction (PPI) Network Construction

The protein-protein interaction (PPI) data of SYP targets and DKD genes were collected from STRING 11.0 (http://string-db.org/). “Homo sapiens” was chosen, and a scoring value >0.9 was selected as the high confidence basis for protein interactions. The common target PPl network files were imported into Cytoscape 3.6.0 and merged to determine the intersection between PPl network and core genes.

### 2.3. Network Construction

To elucidate the role of therapeutic target proteins in gene function and signaling pathways, Gene ontology (GO) and Kyoto Encyclopedia of Genes and Genomes (KEGG) pathway enrichment analyses were performed using R language software. *P* < 0.05 was considered significant.

### 2.4. Animals

Thirty male db/db mice (7 weeks old) and ten wild type (WT) mice (7 weeks old) were purchased from Nanjing University-Nanjing Institute of Biomedicine (license number: SCXK (Su)-2015-0001). The experiment was conducted in the animal laboratory with the barrier room of Hubei University of Chinese Medicine (license number: syxk (HuBei)-2017-0067). The animals were housed in cages in a temperature-controlled room under a 12-hour light/12-hour dark cycle. After adaptive feeding for one week, the mice were randomly divided into three groups: (1) nondiabetes (WT, Con), (2) diabetes model (db/db, Mod), and (3) db/db + treatment (db/db + 6.0 g SYP/kg, SYP). The mice were allowed to feed on standard chow and water. Blood glucose level was tested every four weeks. Each mouse group was raised for 12 weeks. Permission for the animal experiments was granted by the guidelines of the Animal Ethics Committee of Hubei University of Chinese Medicine.

### 2.5. Drugs

SYP (lot number: 20190602) was provided by Hubei Provincial Hospital of Traditional Chinese Medicine, which was located in the Traditional Chinese Medicine Preparation Room of Hubei Provincial Hospital of Traditional Chinese Medicine. SYP is composed of three ingredients in the Chinese Materia Medica (CMM): *Radix Astragali*, *Rhubarb*, and *Herba Epimedii*, at a fixed ratio of 2 :2:1. Anti-Bax (A19684), anti-Bcl-2 (A19693), anti-caspase 3(A19654), anti-Cyto-c (A4912), and anti-*β*-actin (AC026) antibodies were purchased from ABclonal (Wuhan, China).

### 2.6. Measurement of Urinary Protein and Renal Function

Every 4 weeks, the mice were individually housed in metabolic cages for 24 h urine collection. Each urine sample was centrifuged at 4200 rpm for 15 min. The 24 h urinary proteins were determined using a mouse urinary albumin ELISA kit which was purchased from Nanjing Jian Cheng Co. (Nanjing Jian, China). After 12 weeks, the mice were sacrificed for blood collection. The blood was analyzed using an automatic biochemical instrument to determine the renal function.

### 2.7. Hematoxylin & Eosin and Masson's Trichrome Staining

Fresh kidney tissue was fixed in 4% paraformaldehyde and embedded in paraffin. The paraffin blocks of renal tissue were cut to 5 *μ*m thickness. The sections were deparaffinized in xylene and dehydrated in ascending grades of alcohol. After rehydration, the sections were stained with hematoxylin and eosin (HE) and Masson's trichrome. All the images were visualized with a microscope (Nikon, Japan).

### 2.8. TUNEL Staining

The paraffin blocks were dehydrated and deparaffinized, followed by routine staining. After blocking with serum for 30 min, the terminal transferase mediated dUTP nick-end labeling (TUNEL) was used to label the nuclear DNA fragments to detect apoptotic cells. The detection kit was purchased from Servicebio (Wuhan, China) to measure the rate of apoptosis in the db/db mice kidney. After the TUNEL staining, the nuclei were stained with 2-(4-Amidinophenyl)-6-indolecarbamidine dihydrochloride (DAPI) in green. Finally, the slide was sealed and observed. The images were analyzed using a laser scanning confocal microscope. Apoptosis was detected with Image Pro Plus 6.0.

### 2.9. Western Blot Assay

Total protein was extracted from kidney tissue using RIPA buffer (Servicebio, China) and detected with a BCA protein assay kit (Nanjing Jian Cheng Co., China) to determine the concentration of total protein. Protein was separated by SDS-PAGE, electrophoretically transferred to PVDF membranes, and then blocked in 5% nonfat milk. The protein bands were incubated at 4°C overnight with primary antibodies, including Bax, Bcl-2, caspase 3, and Cyto-c, followed by incubation with the secondary antibody for 2 hours at room temperature. Protein expression was detected using the Quantity One analysis system (BioRad, USA) and analyzed with ImageJ. *β*-Actin was used as an internal reference.

### 2.10. Statistical Analyses

Experimental data are presented as mean ± standard deviation. One-way ANOVA test was performed for three groups using GraphPad Prism 8.0 software. *P* < 0.05 was considered to represent a statistically significant difference.

## 3. Results

### 3.1. Target Delineation and Component-Target Network of SYP

**Figure 1:**
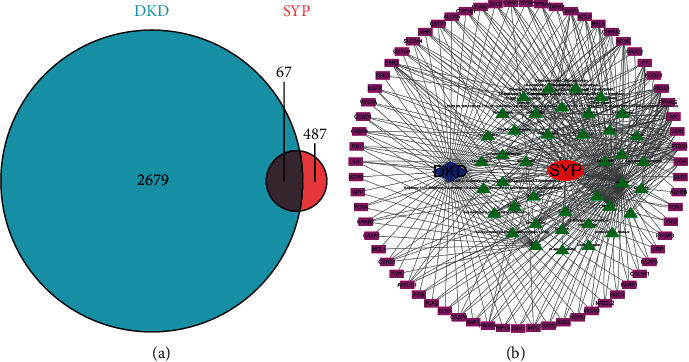
Targets fishing and component-target network of SYP. (a) Targets of both DKD and SYP. (b) Component-target network of SYP. The red ellipse, blue diamond, purple rectangle, and green triangles represent the drug, disease, target genes, and components, respectively; the edges represent the interactions between them.

Drug-related targets (487) were retrieved from the TCMSP database, and 2679 DKD-related target genes were retrieved from the OMIM and GeneCards databases. We then inputted drug and disease targets into Cytospace. Eliminating the redundancy, 67 common targets between DKD and SYP were identified as shown in Figure 1(a). To build a component-target network, the pertinent information of SPY was imported into Cytoscape. As shown in Figure 1(b), the nodes of different colors and shapes indicate the components and potential targets of SYP. The green triangles represent potential targets, purple rectangle indicates disease, blue diamond refers to effective components of SPY, and red ellipse denotes DKD. Thus, the figure shows that the pharmacological effects of Chinese herbal medicine are mediated via multiple components and targets.

### 3.2. Protein-Protein Interaction Network of SYP

**Figure 2:**
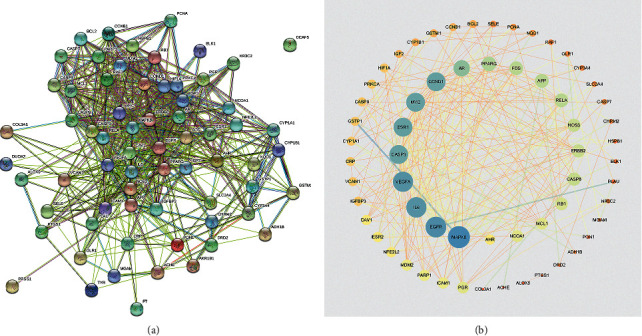
Protein-protein interaction network of SYP. (a) Network nodes represent proteins; edges indicate protein-protein associations. (b) Potential effective targets in DKD.

The common targets were imported into the STRING database to determine their interaction, and a score >0.9 was selected as the basis of high confidence in protein interactions. As shown in Figure 2(a), the interaction network contains 67 nodes and 267 edges. The apoptosis-related proteins among these points and edges are mentioned in Figure 2(b), such as caspase 3, caspase 7, caspase 8, caspase 9, Bax, and Bcl-2. This indicates that apoptosis-related proteins play an important role in renal pathology associated with DKD. In turn, it represents a potential therapeutic target of SYP in DKD.

### 3.3. GO and KEGG Pathway Analysis of SYP

**Figure 3:**
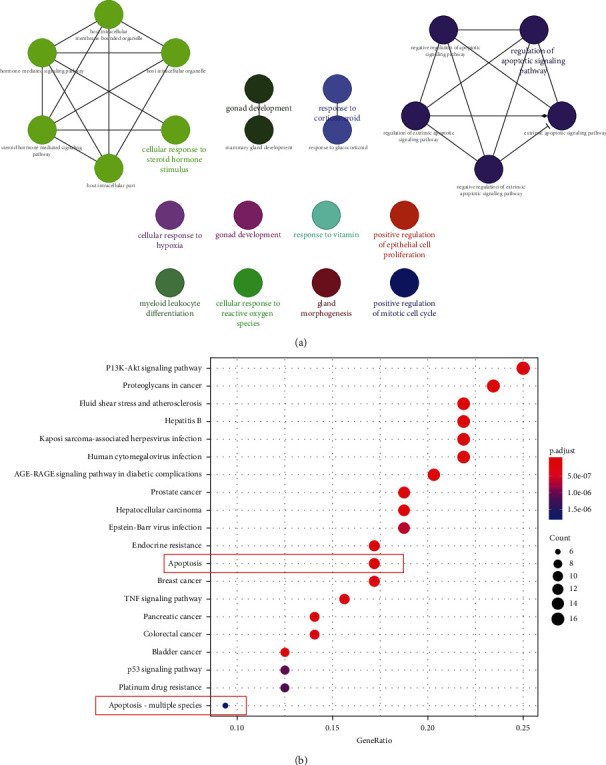
Diagram showing GO and KEGG pathway enrichment following SYP intervention. (a) GO map of putative target genes. (b) KEGG pathway analysis of putative target genes.

In order to delineate the potential therapeutic mechanism of SYP in DKD, GO function and KEGG pathway analyses were performed in R language and Cytoscape. As shown in Figure 3(a), the top 12 functions were enriched by Cytoscape, such as cellular response to steroid hormone stimuli, gonad development, response to corticosteroids, regulation of apoptotic signaling pathway, cellular response to hypoxia, gland development, response to vitamins, positive regulation of epithelial cell proliferation, myeloid leukocyte differentiation, cellular response to reactive oxygen species, glandular morphogenesis, and positive regulation of mitotic cell cycle. As shown in Figure 3(b), two key pathways are strongly related to apoptosis in the top 20 signaling pathways (*P* < 0.01). Multiple signaling pathways were related to apoptosis. The results of PPI showed the proteins related to apoptosis, including CASP3, CASP7, CASP8, CASP9, Bax, and Bcl-2, suggesting a renal protective effect of SYP based on the endpoint index. Next, we will provide animal experimental data to validate the aforementioned signaling pathway.

### 3.4. SYP Improves Renal Function in Db/Db Mice

**Figure 4:**
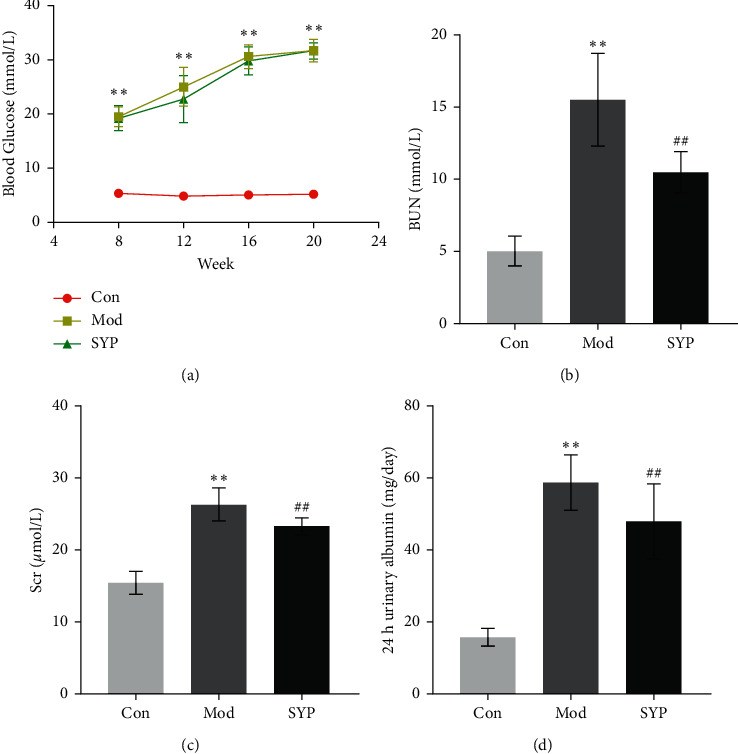
The effect of SYP on blood glucose (a), serum creatinine (b), blood urea nitrogen (BUN) (c), and 24-h urinary albumin (d). Data are expressed as mean ± SD (*n* = 3/group). ^*∗∗*^*P* < 0.01 as compared with the Con group, ^##^*P* < 0.01 as compared with the Mod group. ^△△^*P* > 0.01 as compared with the Mod group.

Previous experiments demonstrated that the 6.0 mg/kg dose of SPY was well-tolerated, without side effects in the SPY group. As shown in Figure 4(a), the blood glucose was higher in both Mod and SYP groups than in the Con group. In addition, no significant difference in blood glucose level was detected between Mod and SYP groups. The levels of serum creatinine, blood urea nitrogen, and 24-h urinary albumin were higher in both Mod and SYP groups than in the Con group as shown in Figures 4(b)–4(d). However, the levels of serum creatinine, blood urea nitrogen, and 24-h urinary albumin were significantly different between SYP and Mod groups. The results indicate that SYP can improve the renal function of db/db mice without decreasing blood glucose levels.

### 3.5. SYP Improves Renal Pathology in Db/Db Mice

**Figure 5:**
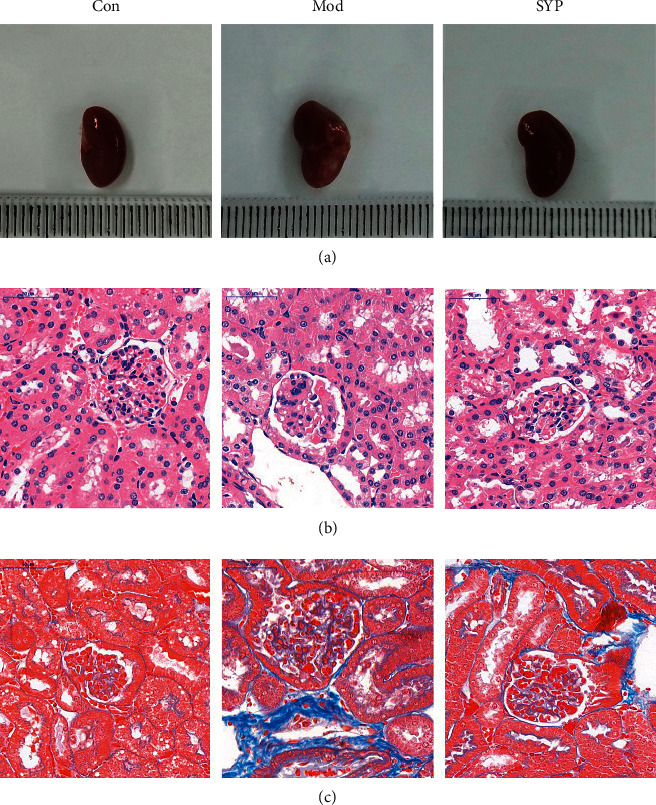
Evaluation of kidney injury via HE and Masson staining in db/db mice treated with SPY. (a) The morphology of kidney in each group. (b) HE staining of kidney in each group. (c) Masson staining of kidney in each group.

Renal fibrosis, which is characterized by excessive deposition of ECM and collagen, is a key factor in the progression of DKD. The aim of this study was to investigate whether SYP reduced the deposition of collagen to improve renal pathology. As shown in Figures 5(a)–5(c), special kidney staining was performed to demonstrate the degree of renal injury in db/db mice. As shown in Figures 5(b) and 5(c), the structure of glomeruli and tubules was clear and complete in the Con group. However, the glomerular basement membrane in the Mod group was thicker than in the Con group. In addition, the collagen deposition was higher in the Mod group than in the Con group. Compared with the Mod group, the thick glomerular basement membrane and collagen deposition were improved significantly in the SYP group, indicating that diabetes induced kidney damage, which was alleviated by SYP.

### 3.6. SYP Attenuated Renal Apoptosis in Mice with DKD

**Figure 6:**
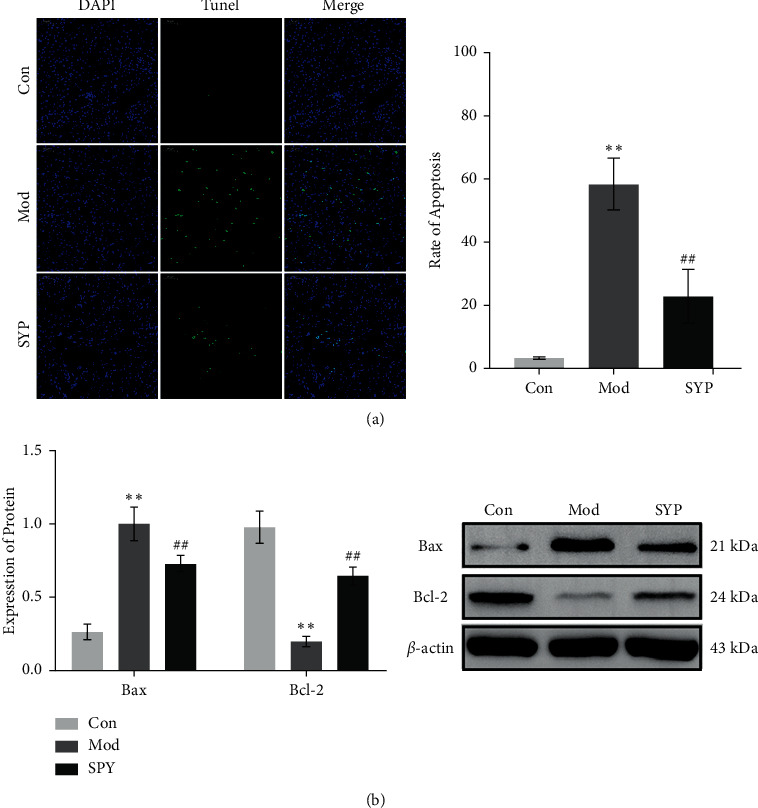
Renal cell apoptosis in db/db mice. (a) Micrographs of TUNEL assay. TUNEL-positive cells are stained green. (b) The expression of Bax and Bcl-2 was detected by Western blot (*n* = 3/group).

Apoptosis is one of the important factors triggering kidney damage in diabetes. The antiapoptotic effects of SYP on renal cells in db/db mice were detected via TUNEL staining. As shown in Figure 6(a), the number of apoptotic renal cells in the Mod group was significantly higher than that in the Con group (*P* < 0.01). As shown in Figure 6(b), the expression of Bax was increased significantly (*P* < 0.01), and the expression of Bcl-2 was decreased significantly (*P* < 0.01). Compared with the Mod group, the number of apoptotic renal cells in the SYP group was reduced significantly. Furthermore, the expression of Bax was decreased significantly (*P* < 0.01), and the expression of Bcl-2 was increased significantly (*P* < 0.01). It indicates that SYP inhibits renal cell apoptosis by reducing the expression of Bax and increasing the expression of Bcl-2.

### 3.7. SYP Inhibits Mitochondrial Injury to Attenuate Kidney Damage in Db/Db Mice

**Figure 7:**
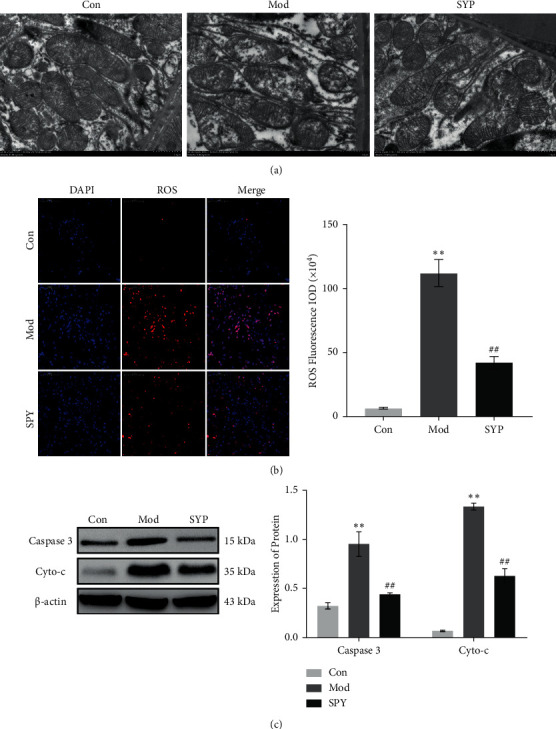
Mitochondrial damage in the kidneys treated with SYP. (a) TEM micrographs. (b) Micrographs of ROS staining. (c) The expression of Cyto-c and caspase 3 was detected by Western blot (*n* = 3/group).

Mitochondria-mediated intrinsic apoptosis is one of the common pathological features in renal damage. As shown in Figure 7(a), we found the mitochondrial cristae were damaged or lost in the Mod group in progressive DKD based on TEM findings. As shown in Figure 7(b), mitochondrial damage released reactive oxygen species (ROS), which induces oxidative stress and apoptosis significantly. As shown in Figure 7(c), the expression of Cyto-c and caspase 3, which are apoptosis-related proteins, increased significantly in the Mod group. Compared with the Mod group, the group treated with SYP showed alleviation of mitochondrial damage and a decrease in the production of ROS. In addition, the expression of Cyto-c and caspase 3 decreased significantly with SYP treatment, which indicates that SYP attenuated renal cell apoptosis by improving the mitochondrial function in db/db mice.

## 4. Discussion

In the past decades [15, 16], the incidence of T2DM has increased rapidly due to the complex interactions between genetic, metabolic, and environmental factors (diet and lifestyle) and hormonal deficiencies. DKD is the most common microvascular complication in DM. Currently, the incidence of DKD is increasing each year, which not only seriously threatens the health in patients but also increases the socioeconomic burden [17].

The efficacy of TCM treatment in DKD has been verified over several hundreds of years clinically. Modern TCM faces significant challenges due to the pharmacological characteristics of multiple components, targets, and channels [13, 18, 19]. The rapid development of bioinformatics, systems biology, and poly-pharmacology has led to innovative applications of network pharmacology in the study of TCM. Network pharmacology was used to analyze the bioactive components and screen corresponding targets. Furthermore, we explored the relationship between the bioactive components and disease to ensure accuracy of the potential targets [20, 21]. We then designed an in vivo experiment to validate the results of network pharmacology analysis.

SYP includes three kinds of medicine: *Radix Astragali*, *Rhubarb*, and *Herba Epimedii*. *Radix Astragali*, *Rhubarb*, and *Herba Epimedii* protect the kidneys by combating the inflammation [22] and oxidative stress [23] and inhibiting apoptosis [24]. The key findings of our study involving network pharmacology analysis and experimental validation in vivo were as follows: (1) sixty-seven predictive targets were identified in TCMSP, GeneCards, and STRING databases. (2) GO and KEGG enrichment analyses showed that the antiapoptotic pathway might be the major mechanism underlying SYP efficacy in DKD. (3) SYP alleviated the renal pathology of the db/db mice and thereby improved the renal function. (4) SYP improved renal cell apoptosis by alleviating mitochondrial damage and enhancing the mitochondrial function.

We first constructed the pharmacological network of SYP and identified 67 predicted targets. Functional enrichment analysis showed that SYP regulated DKD via multiple biological mechanisms such as apoptosis signaling, cell response to hypoxia, gland development, response to vitamins, positive regulation of epithelial cell proliferation, differentiation of myeloid leukocytes, and cellular response to reactive oxygen species. The significantly expressed genes were mainly enriched in apoptosis and apoptosis-multiple species. The proteins of the most meaningful module were mainly related to apoptosis. These results suggested that the antiapoptotic function might be a key mechanism of SYP in the treatment of DKD. Therefore, this study focused on the antiapoptotic mechanism in db/db mice treated with SYP.

Apoptosis is a common pathological feature in individuals diagnosed with DKD, which can be activated via mitochondria-mediated intrinsic [25] and death-receptor-associated extrinsic pathways [19, 26]. DKD also plays a critical role in the progression of chronic kidney disease [27] and renal failure [28, 29]. Studies have suggested that the pathological role of apoptosis may be more pronounced in renal failure due to inflammation [30, 31], necrosis [32], and oxidative stress [33]. Under high glucose [34, 35], the large number of electrons are transferred to the inner mitochondrial membrane, leading to the saturation of electron transport chain and transfer of excess electrons to molecular oxygen, resulting in large amounts of ROS in kidney. In addition, due to the excessive accumulation of ROS, the antioxidant capacity of mitochondria is impaired and the mitochondrial membrane is lost, which in turn decreases the mitochondrial membrane potential, opening the mitochondrial membrane permeability transition pore (mPTP), and release of Cyto-c into the cytoplasm, which ultimately contributes to the aspartic acid-specific cysteine proteolytic enzyme (caspase) cascade [36–38].

In our experimental study, db/db mice were used as the DKD model to determine the renal protective effects of SYP, especially antiapoptotic effects. Our pharmacological investigation showed that SYP treatment improved renal pathology in db/db mice, which was accompanied by decreased serum creatinine, blood urea nitrogen, and 24-h urinary albumin levels. These results indicated that SYP improves renal function by ameliorating renal pathological injury. We then evaluated the antiapoptotic mechanism of SYP in db/db mice kidney. The apoptotic index based on TUNEL staining declined significantly following SYP treatment. Altered cellular apoptosis suggests dysfunctional mitochondria in kidney cells. TEM was used to investigate mitochondrial morphology. The results showed that the mitochondrial numbers increased significantly after treatment with SYP for 12 weeks. In addition, the immunofluorescence showed a marked decrease in ROS in the db/db mice kidney after treatment with SYP for 12 weeks. We also found that the expression of apoptosis-related proteins such as Bax, caspase 3, and Cyto-c declined after treatment with SYP for 12 weeks, and the expression of antiapoptotic proteins such as Bcl-2 increased. These results indicated that SYP treatment decreased apoptosis in the kidney cells of db/db mice by improving the mitochondrial function.

## 5. Conclusion

This study validated 67 predicted targets in SYP corresponding to compounds from three databases and built a detailed PPI network for further bioinformatics analyses. The antiapoptotic effect might be one of key mechanisms of SYP in the treatment of DKD. Furthermore, we used db/db mice as the DKD model and validated the antiapoptotic function of SYP. These data suggest that SYP exhibits antiapoptotic function in vivo, and the mechanism might be mediated via regulation of mitochondrial morphology.

## Data Availability

The original data analyzed in this study are presented in the article.

## Supplementary Materials

Supplementary Materials Western blot-bands: the expression of Bax, Bcl-2, Cyto-c, and caspase 3 was detected by Western blot (*n* = 3/group), and *β*-actin was used as a reference protein.

## References

[1] Williams R., Karuranga S., Malanda B. (2020). Global and regional estimates and projections of diabetes-related health expenditure: results from the international diabetes federation diabetes atlas, 9th edition. *Diabetes Research and Clinical Practice*.

[2] Wheeler D. C., Stefansson B. V., Batiushin M. (2020). The Dapagliflozin and Prevention of Adverse Outcomes in Chronic Kidney Disease (DAPA-CKD) Trial: Baseline Characteristics. *Randomized Controlled Trial*.

[3] Tuttle K. R., Brosius F. C., Cavender M. A. (2021). SGLT2 inhibition for CKD and cardiovascular disease in type 2 diabetes: report of a scientific workshop sponsored by the national kidney foundation. *Diabetes*.

[4] Doshi S. M., Friedman A. N. (2017). Diagnosis and management of type 2 diabetic kidney disease. *Clinical Journal of the American Society of Nephrology*.

[5] Fernandez-Fernandez B., Ortiz A., Gomez-Guerrero C., Egido J. (2014). Therapeutic approaches to diabetic nephropathy--beyond the RAS. *Nature Reviews Nephrology*.

[6] Qin X., Zhao Y., Gong J. (2019). Berberine protects glomerular podocytes via inhibiting drp1-mediated mitochondrial fission and dysfunction. *Theranostics*.

[7] Xiang L., Jiang P., Zhou L. (2016). Additive effect of qidan dihuang grain, a traditional Chinese medicine, and angiotensin receptor blockers on albuminuria levels in patients with diabetic nephropathy: a randomized, parallel-controlled trial. *Evidence-based Complementary and Alternative Medicine*.

[8] Xiaobing L., Chunling N., Wenyu C., Yan C., Zhenzhen L. (2020). Effect of danggui-shaoyao-san-containing serum on the renal tubular epithelial-mesenchymal transition of diabetic nephropathy. *Current Pharmaceutical Biotechnology*.

[9] Zhang H. Y., Tian J. X., Lian F. M. (2021). Therapeutic mechanisms of traditional Chinese medicine to improve metabolic diseases via the gut microbiota. *Biomedicine & Pharmacotherapy*.

[10] Gai Z., Wang T., Visentin M., Kullak-Ublick G. A., Fu X., Wang Z. (2019). Lipid accumulation and chronic kidney disease. *Nutrients*.

[11] Nakagawa T., Yokozawa T., Terasawa K., Nakanishi K. (2003). Therapeutic usefulness of keishi-bukuryo-gan for diabetic nephropathy. *Journal of Pharmacy and Pharmacology*.

[12] He Y., Yue Y., Zheng X., Zhang K., Chen S., Du Z. (2015). Curcumin, inflammation, and chronic diseases: how are they linked?. *Molecules*.

[13] Lu Z., Zhong Y., Liu W., Xiang L., Deng Y. (2019). The efficacy and mechanism of Chinese herbal medicine on diabetic kidney disease. *Journal of Diabetes Research*.

[14] Zou X. R., Zhan L. R., Chen L. (2019). Influence of the Klotho/FGF23/Egr1 signaling pathway on calcium-phosphorus metabolism in diabetic nephropathy and the intervention of Shenyuan granules. *Journal of Biological Regulators & Homeostatic Agents*.

[15] Hashiesh H. M., Meeran M. N., Sharma C., Sadek B., Kaabi J. A., Ojha S. K. (2020). Therapeutic potential of *β*-caryophyllene: a dietary cannabinoid in diabetes and associated complications. *Nutrients*.

[16] Shore-Lorenti C., Brennan S. L., Sanders K. M., Neale R. E., Lucas R. M., Ebeling P. R. (2014). Shining the light on Sunshine: a systematic review of the influence of sun exposure on type 2 diabetes mellitus-related outcomes. *Clinical Endocrinology*.

[17] de Vries J. K., Levin A., Loud F., Adler A., Mayer G., Pena M. J. (2018). Implementing personalized medicine in diabetic kidney disease: stakeholders’ perspectives. *Diabetes, Obesity and Metabolism*.

[18] Qin X., Jiang M., Zhao Y. (2020). Berberine protects against diabetic kidney disease via promoting PGC-1*α*-regulated mitochondrial energy homeostasis. *British Journal of Pharmacology*.

[19] Warren A. M., Knudsen S. T., Cooper M. E. (2019). Diabetic nephropathy: an insight into molecular mechanisms and emerging therapies. *Expert Opinion on Therapeutic Targets*.

[20] Li S., Zhang B. (2014). Traditional Chinese medicine network pharmacology: theory, methodology and application. *Chinese Journal of Natural Medicines*.

[21] Zhang R., Zhu X., Bai H., Ning K. (2019). Network pharmacology databases for traditional Chinese medicine: review and assessment. *Frontiers in Pharmacology*.

[22] Qi M. Y., He Y. H., Cheng Y. (2021). Icariin ameliorates streptozocin-induced diabetic nephropathy through suppressing the TLR4/NF-*κ*B signal pathway. *Food & Function*.

[23] Hu R., Wang M., Liu L. (2020). Calycosin inhibited autophagy and oxidative stress in chronic kidney disease skeletal muscle atrophy by regulating AMPK/SKP2/CARM1 signalling pathway. *Journal of Cellular and Molecular Medicine*.

[24] Tian R., Wang P., Huang L. (2020). Sanqi oral solution ameliorates renal ischemia/reperfusion injury via reducing apoptosis and enhancing autophagy: involvement of ERK/mTOR pathways. *Frontiers in Pharmacology*.

[25] Jiang H., Shao X., Jia S. (2019). The mitochondria-targeted metabolic tubular injury in diabetic kidney disease. *Cellular Physiology and Biochemistry*.

[26] Oe Y., Miyazaki M., Takahashi N. (2021). Coagulation, protease-activated receptors, and diabetic kidney disease: lessons from eNOS-deficient mice. *Tohoku Journal of Experimental Medicine*.

[27] Colhoun H. M., Marcovecchio M. L. (2018). Biomarkers of diabetic kidney disease. *Diabetologia*.

[28] Bakris G. L., Agarwal R., Anker S. D. (2020). Effect of finerenone on chronic kidney disease outcomes in type 2 diabetes. *New England Journal of Medicine*.

[29] Kato M., Natarajan R. (2019). Epigenetics and epigenomics in diabetic kidney disease and metabolic memory. *Nature Reviews Nephrology*.

[30] Pérez-Morales R. E., Del Pino M. D., Valdivielso J. M., Ortiz A., Mora-Fernández C., Navarro-González J. F. (2019). Inflammation in diabetic kidney disease. *Nephron*.

[31] Reidy K., Kang H. M., Hostetter T., Susztak K. (2014). Molecular mechanisms of diabetic kidney disease. *Journal of Clinical Investigation*.

[32] Chen Y., Lee K., Ni Z., He J. C. (2020). Diabetic kidney disease: challenges, advances, and opportunities. *Kidney Disease*.

[33] Jha J. C., Banal C., Chow B. S., Cooper M. E., Jandeleit-Dahm K. (2016). Diabetes and kidney disease: role of oxidative stress. *Antioxidants and Redox Signaling*.

[34] Miyamoto S., Zhang G., Hall D. (2020). Restoring mitochondrial superoxide levels with elamipretide (MTP-131) protects db/db mice against progression of diabetic kidney disease. *Journal of Biological Chemistry*.

[35] Afsar B., Hornum M., Afsar R. E. (2021). Mitochondrion-driven nephroprotective mechanisms of novel glucose lowering medications. *Mitochondrion*.

[36] Yuan S., Liu X., Zhu X. (2018). The role of TLR4 on PGC-1*α*-mediated oxidative stress in tubular cell in diabetic kidney disease. *Oxidative Medicine and Cellular Longevity*.

[37] Zhan M., Usman I. M., Sun L., Kanwar Y. S. (2015). Disruption of renal tubular mitochondrial quality control by Myo-inositol oxygenase in diabetic kidney disease. *Journal of the American Society of Nephrology*.

[38] Liu L., Zhang L., Zhao J. (2020). Tumor necrosis factor receptor-associated protein 1 protects against mitochondrial injury by preventing high glucose-induced mPTP opening in diabetes. *Oxidative Medicine and Cellular Longevity*.

