# Supplementary figures and images for: Renal Protective Mechanisms of Shenyuan Particle in Db/Db Mice: A Study Based on Network Pharmacology
Source: Evid Based Complement Alternat Med. 2022 Jun 14;2022:9579179. doi: 10.1155/2022/9579179 (PMC9213133; doi:10.1155/2022/9579179)

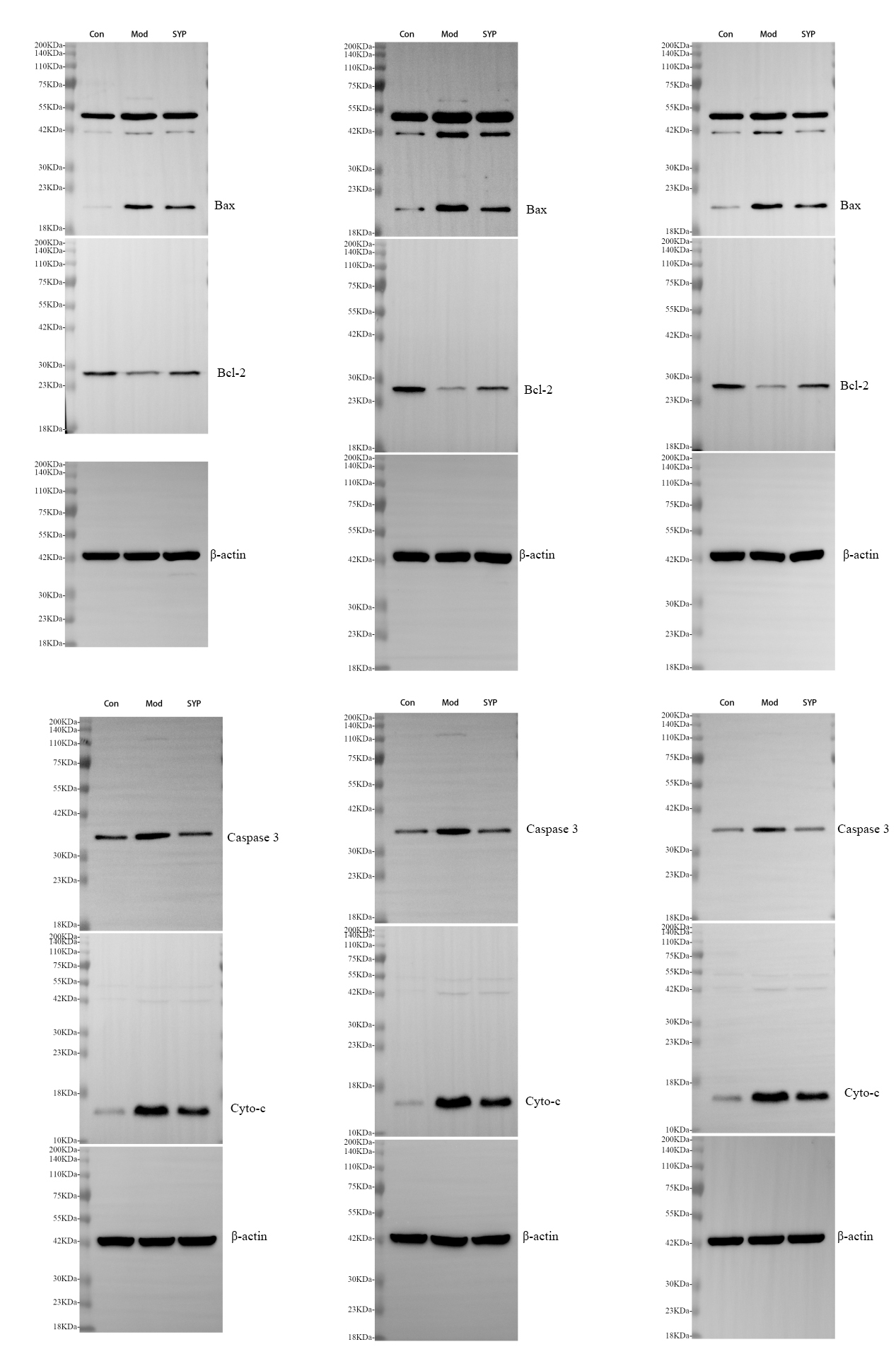

Supplement: Supplementary Materials — Western blot-bands: the expression of Bax, Bcl-2, Cyto-c, and caspase 3 was detected by Western blot (n = 3/group), and β-actin was used as a reference protein. [file 9579179.f1.jpg]
